# The Epidemiology of Youth Sport-Related Shoulder Injuries: A Systematic Review

**DOI:** 10.1155/2022/8791398

**Published:** 2022-08-23

**Authors:** Eric S. Gibson, Alexis Cairo, Anu M. Räisänen, Colleen Kuntze, Carolyn A. Emery, Kati Pasanen

**Affiliations:** ^1^Sport Injury Prevention Research Centre, Faculty of Kinesiology, University of Calgary, Calgary, Canada; ^2^Department of Physical Therapy Education, College of Health Sciences - Northwest, Western University of Health Sciences, Lebanon, Oregon, USA; ^3^Alberta Children's Hospital Research Institute, University of Calgary, Calgary, Canada; ^4^O'Brien Institute for Public Health, University of Calgary, Calgary, Canada; ^5^Hotchkiss Brain Institute, University of Calgary, Calgary, Canada; ^6^Community Health Sciences, Cumming School of Medicine, University of Calgary, Calgary, Canada; ^7^Department of Pediatrics, Cumming School of Medicine, University of Calgary, Calgary, Canada; ^8^McCaig Institute for Bone and Joint Health, University of Calgary, Calgary, Canada; ^9^Tampere Research Center of Sports Medicine, UKK Institute, Tampere, Finland

## Abstract

**Background:**

Youth around the globe place their shoulders at risk for injury when participating in sports. Shoulder injuries may vary in severity, produce the potential for time-loss from sport, and result in functional disability. We sought to explore sport-related shoulder injuries in youth by identifying injury rates, risk factors, injury mechanisms, and injury prevention strategies.

**Methods:**

All relevant full-text articles were identified by searching MEDLINE, EMBASE, CINAHL, Sport Discus, and the Cochrane Controlled Trials Registry. No date restrictions were used. All full-text studies reporting original research describing sport-related shoulder injury among female and/or male youth from 5 to 18 years old were included.

**Results:**

Of 3,889 studies screened, 97 described shoulder injury in youth sports. Shoulder injuries were identified in 24 unique sports. The median seasonal prevalence of shoulder injury was 10.9% (range 1.2–28.2%). The most common injury mechanisms identified were contacted with another player, contact with the playing environment, and falling to the ground. Risk factors for shoulder injury identified were side-to-side strength imbalances, weak external rotator muscles, and scapular dyskinesia. One study evaluated a successful training strategy to prevent shoulder injuries, but two other interventions demonstrated no effect.

**Conclusions:**

Sport-related shoulder injuries are prevalent among youth athletes. Injury risk factors identified included modifiable intrinsic factors such as strength, range of motion, and training load. The most common injury mechanism was direct contact with either another person or an object in the playing environment. Innovative shoulder-specific strategies are needed to reduce shoulder injuries in this population. Trial Registration: PROSPERO ID: CRD42020189142.

## 1. Introduction

Each year, millions of youth around the world participate in sports that place them at potential risk for shoulder injury [1, 2]. Biomechanically, the shoulder has the greatest mobility of any joint in the body, leading to an increased injury risk due to the inherent lack of stability [3]. The risk of injury is exacerbated when the arm is placed in an overhead or outstretched position [4, 5]. Common injuries include acute and repetitive muscle/tendon strains or tears and ligament sprains or ruptures; rotator cuff-related disorders; labral pathology; acromioclavicular joint separation; dislocation of the humerus from the glenohumeral joint; fractures of the clavicle, humerus, or scapula; bruising; generalized and/or complex pain syndromes; and tendinopathies [5–7]. Shoulder injuries may vary in severity and lead to time-loss from sport and functional disability immediately or later in life. Beyond time-loss from sport-specific training and competition activities, acute and chronic shoulder injuries are characterized by experiences of pain, weakness and/or instability with reaching, pushing, or pulling movements during day-to-day activities [8], and interrupted sleep due to night pain [9]. Considering the functional impacts of injury, an improved understanding of what characterizes and results in sport-related shoulder injury is needed. While it would be ideal if preventative measures were taken to prevent sport-related shoulder injuries from occurring in young athletes, what characterizes an effective intervention has yet to be shown. If a one-size-fits-all approach could be taken to prevent shoulder injuries would be most appropriate and should also be considered. To date, however, in the youth injury prevention literature, there has been a continued focus on other musculoskeletal conditions (related to the hip, knee, and ankle), as well as a concussion. The rationale for this review is therefore to map the literature that has been previously been published on sport-related shoulder injuries. The primary objective of this systematic review is to explore the epidemiology of sport-related shoulder injuries in youth by identifying the prevalence, incidence rates, risk factors, injury mechanisms, and injury prevention strategies that have undergone scientific investigation.

## 2. Methods

### 2.1. Searches

We identified all potentially relevant articles by searching MEDLINE, EMBASE, CINAHL, Sport Discus, and the Cochrane Controlled Trials Registry. Articles indexed from database inception up until August 2021 were searched in each database. These electronic databases were searched using three comprehensive search concepts (Table1).

We combined the results using the Boolean operator “AND.” The search was enhanced by a hand search of the references of any existing systematic reviews identified in our search. Full-text articles not available or not written in English were excluded.

### 2.2. Study Inclusion and Exclusion Criteria

All full-text studies reporting original research that described sport-related shoulder injury among female and/or male youth from 5 to 18 years old using any study design were included. Studies were excluded if they were synthesis or review papers; were small case-series (less than 10 participants) or single case reports; if the participants included more than 10% adult participants; or if the full text was not available for review (or not available in English). Inter-rater agreement was evaluated based on the first 50 records retrieved and imported into Microsoft Excel. An acceptable level of agreement was set at equal to or greater than 80% [10]. Two authors (ESG and AC) independently screened all identified titles and abstracts for eligibility using Covidence and disagreements were resolved by a third author (AMR). If the title and abstract provided insufficient information to determine eligibility, the full text was reviewed for inclusion. Full-text articles were then screened for eligibility by the same two authors (ESG and AC). Any disagreements regarding inclusion following full-text review screening were resolved by author consensus (ESG, AC, AMR).

### 2.3. Study Quality Assessment

Study quality was assessed using the Downs and Black checklist out of a maximum possible score of 33 [11]. The level of evidence was assessed in line with the Oxford guidelines (Level I-VII) [12]. These are further defined as follows: Level 1 (systematic review); Level II (randomized controlled trial); Level III (controlled trials, no randomization); Level IV (case-control or cohort); Level V (synthesis of descriptive or qualitative studies); Level VI (single descriptive or qualitative studies); and Level VII (opinion pieces/expert authority).

### 2.4. Data Extraction Strategy

Data extraction was independently completed in a Microsoft Excel spreadsheet by one of the two authors for each of the included papers. A data extraction template was pilot tested and inter-rater agreement was evaluated a priori. The following data were extracted for each study: author, publication year, geographic location of the study, sample size, primary sport, level of competition, length of study, how shoulder injury was operationalized, injury type (acute/chronic), incidence and/or prevalence of a sport-related shoulder injury, risk factors for sport-related shoulder injury, description of sport-related shoulder injury mechanisms observed, and characteristics of any injury prevention modality that was tried.

## 3. Results

### 3.1. Review Statistics

**Figure 1:**
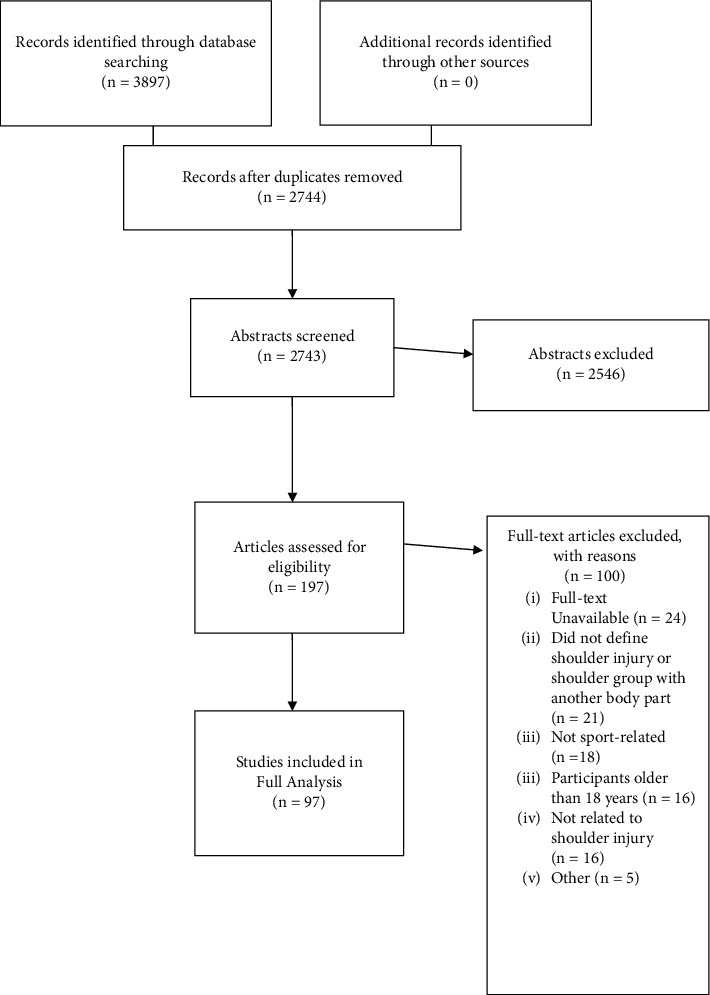
PRISMA flow diagram for study selection.

Figure 1 outlines the process of study selection in accordance with the PRISMA standards for the reporting of systematic reviews. Following the screening process described above, 97 studies that evaluated sport-related shoulder injuries among youth were found and selected for inclusion and data extraction [13–107]. The agreement between the reviewers following full-text screening was 85.2%.  Table 2 describes the included studies (stratified by sport) and Table 3 describes the worldwide geographic distribution of the studies. Study designs varied from randomized controlled trial (RCT; *n* = 3), cluster randomized controlled trial (*n* = 1), prospective cohort (*n* = 58), historical cohort (*n* = 9), cross-sectional (*n* = 25), and case series (*n* = 1).

### 3.2. Shoulder Injury Prevalence and Incidence Rates

Among the studies included in this review, 78 reported shoulder injuries in 24 unique youth sports. The reported prevalence estimates for shoulder injury from the included studies are reported in Table 4. The median seasonal prevalence of shoulder injury was 10.9% (range 1.2–28.2%) across 41 studies. Season length varied across sports, however, ranged from 6 weeks to 12 months. Shoulder injury incidence rates (IR) are summarized by sport in Table 5. Baseball was the sport with the highest shoulder IR with up to 17.6 injuries per 1000 athletic exposures (AEs) [26] and the lowest rate of shoulder injury was observed in girl's lacrosse at 0.10 per 1000 AEs [72]. Shoulder injuries were more common in competition than in training/practice (Table 6).

### 3.3. Shoulder Injury Mechanisms

Three studies included in this review described the mechanisms of acute shoulder injuries. These studies were conducted among hockey (*n* = 8228) and rugby players (*n* = 294 and *n* = 378) [31, 45, 50]. The most common shoulder injury mechanisms were direct contact with another player in rugby (75.9%) and then contact with the boards (28.1%), contact with another player (18.7%), or a fall on the ice (16.6%) on ice hockey.

### 3.4. Risk Factors for Shoulder Injury

Sixteen studies included in this review reported on risk factors for shoulder injuries in youth sports populations [13, 18, 21, 36, 50, 65, 67, 70, 84–86, 93, 103, 104, 107]. Of the studies that identified a risk factor for shoulder injury, the sports represented included baseball/softball, (*n* = 7); handball, (*n* = 3), rugby (*n* = 1); ice hockey, (*n* = 1); mixed high school sports, (*n* = 1); cricket (*n* = 1); swimming (*n* = 1); and tennis (*n* = 1). Modifiable risk factors identified for shoulder injury were shoulder external and internal rotation muscle weakness, scapular dyskinesia, side-to-side strength imbalances, increasing training load by more than 60% relative to the previous month, and playing position [13, 18, 21, 36, 50, 65, 67, 70, 84–86, 93, 103, 107]. Male sex was reported as a nonmodifiable risk factor for a shoulder injury in one study [21].

Based on seven baseball studies, the most common observation was that pitchers were most likely to experience an injury to the throwing shoulder [70, 84, 86]. Two studies noted that side-to-side differences in horizontal adduction and internal rotation weakness as well as reduced range of motion (ROM) were associated with greater shoulder injury prevalence [70, 84]. This was however contradicted by Tyler et al. (2014), who found internal rotation ROM was not associated, but rather supraspinatus muscle weakness led to more injuries throughout a competitive season. Scapular dysfunction was also noted to be common in high school baseball players but was not associated with shoulder injury prevalence [67].

### 3.5. Injury Prevention Strategies

Three RCTs evaluated the utility of short, shoulder-specific training programs. One of these studies demonstrated a reduction in shoulder injury rate [80]. Sakata et al. focused on upper and lower extremity ROM, as well as scapular function in 9 to 11-year-old baseball players [80]. Following participation, the intervention group had a lower shoulder and elbow injury incidence rate (1.7 injuries/1000 AEs) than the control group (3.1 injuries/1000 Aes), or an estimated 48.5% reduction in shoulder injury incidence compared to the control group. Two interventions in handball were also studied but did not prove effective in their goal to reduce risk factors for a shoulder injury. The interventions trialed were an 8-week external rotation-focused strength training program, and the Oslo Sports Trauma Research Center shoulder injury prevention program [105, 106].

### 3.6. Study Quality Assessment

Of the 97 studies, three studies had level II evidence, four had level III evidence, 46 had level IV evidence, five had level V evidence, and 39 had level VI evidence. The median quality score was 10 (range 7–24) out of a maximum possible score of 33 on the Downs and Black checklist. It is noted that the maximum score possible for an observational study was 31. A detailed assessment of study quality can be found in Table 7.

## 4. Discussion

This review included 97 studies that evaluated sport-related shoulder injury in youth sports. While most studies sought to describe injury rates in their given sport, we were also able to identify sixteen studies that described risk factors, as well as the mechanisms for shoulder injuries in three studies. One study evaluated successful injury prevention strategies, but two other studies found their interventions had no effect on the occurrence of shoulder injuries.

The median seasonal prevalence of shoulder injury was 10.9% (range 1.2–28.2%; Table 4). Season length varied across the sports observed, ranging from 6 weeks to 12 months. This serves to suggest that shoulder injury is common across a variety of youth sports and measures to reduce future injuries merit consideration. The shoulder injury rates observed in this review were also higher in competition (range 0.05–2 per 1000 AEs) than in practice/training (range 0.009–0.25 per 1000 AEs). This phenomenon remained true across all of the studies that reported an injury rate that was stratified by session type. This observation may serve to inform future injury prevention strategies by emphasizing the importance of reducing competition injuries as a research priority. This is especially interesting, as athletes typically spend more time participating in training (more repetitive movements) than the competition (more intense exposure).

Three studies investigated the mechanisms for acute shoulder injury [31, 45, 50]. The most common injury mechanisms among the studies included in this review were contact related and resulted in acute/traumatic injury. This was observed in hockey where injuries were related to contact with another player or contact with the boards [31]. In rugby, injuries were most commonly observed in contact with another player [45] and usually due to tackling [50]. While contact injuries are also commonly seen in adult athletes, adult injuries are more often related to overuse and repetitive motions. Previous studies have specifically found that overuse injuries may account for up to 36% (95% CI: 32–39%) of shoulder injuries among adult athletes [23]. Notwithstanding, the occurrence of repetitive shoulder injury may be less common in youth, but further research is needed to describe the rates and mechanisms.

Of the studies identified in this review relevant to injury mechanisms, it is unique that they relate to two contact/collision sports (ice hockey and rugby). While shoulder injuries do appear to occur commonly in these sports, heightened shoulder injury rates were also observed in noncontact, repetitive motion sports such as baseball, softball, and tennis by this review. This further outlines the importance of formulating unique injury prevention strategies in youth.

The most common risk factors identified for youth shoulder injury were shoulder external and internal rotation muscle weakness, ROM deficits, side-to-side strength imbalances, and playing position (specific to pitchers in baseball). Boys may also be more likely to suffer a shoulder injury than girls. Considering the consistently higher injury rates in exposure to competition compared to practice observed in this review, competition play itself may also be a risk factor for a shoulder injury that merits further research (Table 6). With consideration of risk factors identified in contact sports, it was observed in ice hockey that heavier players (>160 pounds) experienced double the risk of shoulder injury than those who fell under this threshold [36]. In rugby, playing position was associated with 1.8 times the odds of a shoulder injury for those in the “front row” [50]. With respect to the studies that identified risk factors in handball, players with internal and external rotation weakness were associated with higher injury rates [13, 18, 65]. Scapular dyskinesia at baseline was also observed as a risk factor for shoulder injury with moderate training load increases (20–60% over the previous month) [66]. Handball studies also represented the only sport in which training load monitoring was investigated. Participation increases of >60% compared to the previous 4-weeks were specifically associated with an increased shoulder injury rate [65]. Further study of training load across various sporting activities and contexts may be a promising avenue for researchers to enhance their understanding of injury risk factors and should be considered for future studies.

Three studies sought to evaluate targeted shoulder injury prevention programs among youth [80, 105, 106]. These were high-quality studies (RCT), where the prevention modality sought to reduce the number of shoulder injuries observed in baseball or handball. Further study is still needed, however, as one study evaluated successful injury prevention strategies [80, 104], but two other studies found their interventions had no preventative effect on injury risk factors [105, 106]. Sakata et al. found their intervention was successful in reducing injuries in a noncontact throwing sport, meaning that such an intervention may or may not be appropriate in other sporting environments (namely, contact/collision sports like ice hockey, handball, rugby, or football). As the two unsuccessful shoulder injury prevention programs observed were designed to target shoulder injury risk factors, this serves to highlight the complexity and need for future innovative studies of this caliber to test innovative prevention strategies across all sport types aiming to reduce shoulder injuries more broadly.

An important knowledge gap relates to the generalizability and reproducibility of potential strategies to minimize injury risk. Given the ultimate goal of reducing shoulder injuries through coherent management of modifiable risk factors, this review assists by describing the rates in which they occur in various sporting populations. Design and study of the injury prevention protocols across various sports and age groups would provide important insights as to whether an intervention is worthy of broader application. A second knowledge gap relates to the ability of an injury prevention strategy to work effectively. Ideally, an intervention would be (1) simple enough for youth and coaches to understand, (2) quick and easy to complete, and (3) produce a measurable reduction in injury rates. Economically, in order to justify the adoption of injury prevention modalities, benefits must also be both feasible and accessible to the average youth athlete. In general, simpler interventions that require little equipment/preparation are more likely to be useful on an ongoing basis to all youth who could benefit. Further research would also serve to inform if a universal approach to shoulder injury prevention is appropriate. Logically, it does appear that sport-specific differences in injury rates, injury mechanisms, and injury risk factors do exist, so interventions likely need to be customized to a given sporting group's circumstances.

A major strength of this study relates to the systematic nature of the literature search, data extraction, and quality assessment. We were able to extensively search several relevant databases with a similar strategy, identify sources of evidence that have undergone previous scientific investigation, and synthesize the results in one place. We were also able to assess the overall quality of the literature, by considering sources of bias and the level of evidence associated with each paper.

An overarching limitation of this study was related to the quality of the included studies. Most of the studies were assigned a level of evidence score of 4 or lower (out of 7; *n* = 89/97). With respect to the Downs and Black checklist, a median quality score was 10 out of a maximum possible score of 33 (range 7–24) was observed. Heterogeneity of study designs and data collection protocols limited our ability to draw direct comparisons both between sports and even within sports due to the variation in data reported. This was complicated in two ways. First, several studies evaluated samples that participated in various sports and pooled them together when completing the formal analysis, so sport-specific estimates could not always be deduced. Second, several of the included studies focused on a specific type of shoulder injury (e.g., dislocation, clavicle fracture), meaning that the count of youth shoulder injuries may have been underestimated. This overall low quality of included studies unfortunately limits the generalizability of the conclusions that can be drawn from our results. Considering, however, that such little, high-quality work (i.e., studies with minimal sources of bias) has been done previously in consideration of youth sport-related shoulder injuries, any synthesis is an addition to the literature. Injury definition was another methodological limitation of this study. There was significant variability in how injuries were described, with some studies using a time-loss definition, some using any shoulder complaints, and others using emergency room visits or hospital records. With this consideration, it is just not feasible to separate analysis into acute and repetitive injuries (especially since the etiology of injury varies). Looking critically towards the evaluation of injury incidence in this review, many studies described injuries per athletic exposure. While this may still be a valuable metric, this does not account for an athlete's time exposure to their sport. As variability in length of session activity may exist, this limits our ability to assess and compare shoulder injury incidence rates.

## 5. Conclusion

The sport-related shoulder injury is prevalent among youth athletes. It appears that some identified injury risk factors may be modifiable (e.g., external and internal rotation muscle weakness, scapular dyskinesia, side-to-side strength imbalances, increasing training load by more than 60% relative to the previous month, and playing position). The most common mechanism of acute shoulder injury was a direct contact with either another person (during tackling or body checking) or contact with an object in the playing environment. This review was, however, limited by the quality of the literature included (a median quality score of 10 out of 33). This suggests the need for further robust study of shoulder injury rates, risk factors, and injury mechanisms, as well as innovative solutions to reduce the burden of youth shoulder injury. This serves to underline the importance of designing and evaluating shoulder-specific injury prevention strategies in an effort to reduce sport-related shoulder injury in youth.

## Figures and Tables

**Table 1:** Search strategy used for the MEDLINE database.

#	Searches
1	exp Adolescent/
2	(Youth^*∗*^ or teen^*∗*^ or child^*∗*^ or adolescent^*∗*^ or juvenile^*∗*^).tw, kf.
3	Pediatric.tw, kf.
4	exp Pediatrics/
5	(student^*∗*^ or varsity or high school or junior high) ADJ2 (athlet^*∗*^ or sport^*∗*^).tw, kf.
6	(boy or boys or girl or girls).tw, kf.
7	OR/1-6
8	exp Shoulder Injuries/
9	shoulder ADJ2 injur^*∗*^.tw, kf.
10	Shoulder.tw, kf.
11	exp Athletic Injuries/ or exp Joint Dislocations/ or exp Sprains/ or Strains/ or exp Fractures, Bone/
12	(dislocate^*∗*^ or fracture^*∗*^ or labral or labrum or subacromial or rotator cuff^*∗*^ or acromioclavicular or sprain^*∗*^ or strain^*∗*^ or scapulothoracic or clavicle or separate^*∗*^OR instability or coracoclavicular or pathology or sternoclavicular or impingement or scapula).tw, kf.
13	10 AND 11
14	10 AND 12
15	8 OR 9 OR 13 OR 14
16	exp Sports/ or exp Basketball/ or exp Baseball/ or exp Soccer/ or exp Hockey/ or exp Football/ or exp Martial arts/ or exp Golf/ or exp Wrestling/ or exp Volleyball/ or exp Swimming/ or exp Skiing/ or exp Racquet sports/ or exp Snow Sports/
17	(sport^*∗*^ or basketball or baseball or softball or soccer or hockey or football or netball or net ball or boxing or mixed martial arts or kickboxing or kick boxing or karate or judo or golf or wrestling or volleyball or swimming or diving or skiing or ski or skating or curling or snow sport^*∗*^ or snowboarding or curling or racquet sport^*∗*^ or racquetball or racquet ball or tennis or ping pong or squash or badminton or weightlifting or weight lifting or powerlifting or canoeing or kayaking or surfing or sailing or climbing or bouldering or ice climbing or gymnastics or trampoline or cycling or mountain biking or BMX or scootering or rollerblading or roller blading or skate boarding or skateboarding or athletics or cheerleading or lacrosse of cricket or ringette or rodeo or rugby or equestrian or water polo or handball or hand ball or team handball or team hand ball).tw, kf.
18	16 OR 17
19	7 AND 15 AND 18
20	Limit 19 to English language

**Table 2:** Worldwide distribution of included studies.

Continent	Total studies (^#^)
North America	63
Europe	21
Oceania	6
Africa	4
Asia	3

**Table 3:** Included studies stratified by sport.

Sporting Population	Total Studies, *n* (%)	Studies (First Author)
Baseball	15 (15)	Arnold 2019; Collins 2008 (1); Fleisig 2011; Hibberd 2018; Krajnik 2010; Myers 2013; Oyama 2017; Register-Mihalik 2012; Sakata 2019; Saper 2018; Shanley 2011 (1); Shanley 2011 (2); Shitatra 2017; Tyler 2014; Wasserman 2019 (1)
Rugby	12 (12)	Archbold 2017; Barden 2018; Collins 2008 (2); Dyer 2019; Haesler 2010; Hodhody 2016; Junge 2004; Kawasaki 2014; Sewry 2018; Tee 2019; Vijam 2015; Yard 2006 (1)
Hockey	8 (8)	Deits 2010; Emery 2006; Finke 1988; Lynall 2018; Matic 2015; Polites 2014; Tuominen 2017; Yard 2006 (2)
Softball	7 (7)	Krajnik 2010; Oliver 2019; Shanley 2011 (1); Shanley 2011 (2); Smith 2015; Snyder-Valier 2020; Wasserman 2019 (2)
Handball	7 (7)	Achenbach 2020; Asker 2020; Asker 2018; Fredriksen 2020; Fredriksen 2021; Moller 2017; Olsen 2006
Football	6 (6)	Badgley, 2013; Culpepper 1983; Dompier 2007, Kerr 2018 (3); Morris 2017; Volpi 2003
Lacrosse	5 (5)	Hinton 2005; Pierpoint 2019 (1); Pierpoint 2019 (2); Warner 2018; Yard 2006 (2)
Soccer	4 (4)	DiStefano 2018; Jacobs 2012; Junge 2004; Kerr 2018 (2)
Volleyball	4 (4)	Kerr 2018 (1); Miranda 2015; Pollard 2011; Reeser 2015
Martial arts/Wrestling	4 (4)	Frey 2019; Kroshus 2018; Pasque 2000; Yard 2007
Basketball	3 (3)	Allen 2019; Clifton 2018 (1), Clifton 2018 (2)
Other/Multiple sports (>3)	22 (23)	Bonza 2009; Clarsen 2015; Currie 2016; Darrow 2009; Drigny 2020; Forrest 2020; Geischeit 2019; Gamage 2019; Hirschhorn 2018; Horobeanu 2019; Hutchinson 1995; Hjelm 2010; Kalo 2020; Kerr 2017; Kraeutler 2018; McCarthy 2019; Rauh 2007; Robinson 2014Ruedl 2012; Schneuer 2018; Swenson 2009; Wood 2010

**Table 4:** Reported shoulder injury prevalence from included studies. Sport-specific seasonal shoulder injury prevalence is reported in descending order. If not stated otherwise, the sample population includes both girls and boys.

Author	Population	Sport-specific seasonal prevalence
Asker	Girls handball	28.2%
Smith	Softball	24.5%
Pasque	Boys wrestling	24.0%
Matic	Ice hockey	20.6%
Dyer	Rugby union	19.9%
Yard	Taekwondo	19.1%
Kawasaki	Boys rugby	17.4%
Emery	Ice hockey	17.0%
Kroshaus	Boys wrestling	16.9%
Sewry	Rugby	15.9%
Morris	Football	15.0%
Snyder-Valier	Softball	14.2%
Yard	Rugby	14.1%
Deits	Ice hockey	13.8%
Culpepper	Boys football	13.3%
Collins	Rugby	12.8%
Kerr	Boys football	12.0%
Polites	Ice hockey	11.3%
Yard	Hockey	11.1%
Miranda	Volleyball	11.0%
Darrow	Mixed sports	10.9%
Wasserman	Boys baseball	10.8%
Pierpoint	Boys lacrosse	10.8%
Ruedl	Mixed sports	9.9%
Reeser	Girls volleyball	8.3%
Hjelm	Tennis	8.0%
Schneur	High school sports	7.7%
Vijam	Girls touch rugby	7.7%
Kerr	Boys mixed sports	7.6%
Kerr	Girls volleyball	6.9%
Hirschorn	Mixed sports	6.8%
Gamage	Cricket	6.1%
Kerr	Girls mixed sports	5.6%
Dompier	Football	5.5%
Yard	Karate	4.4%
Olsen	Handball	4.0%
Wasserman	Girls softball	3.6%
Horobeanu	Squash	3.3%
Kerr	Boys soccer	2.1%
Yard	Judo	1.9%
Pierpoint	Girls lacrosse	1.2%

**Table 5:** Shoulder injury incidence rates reported in included studies. Injury incidence rates by athletic exposure (AE) have been standardized to 1 000 AEs and reported in descending order. If not stated, the sample population includes both girls and boys.

Author	Population	Overall	Denominator
Collins	Baseball	17.6	1000 AEs
Snyder-Valier	Softball	1.07	1000 AEs
Myers	Baseball	1	1000 AEs
Hinton	Boys lacrosse	0.24	1000 AEs
Krajnik	Baseball	0.172	1000 AEs
Saper	Baseball	0.139	1000 AEs
Oliver	Softball	0.114	1000 AEs
Hinton	Girls lacrosse	0.1	1000 AEs
Krajnik	Softball	0.1	1000 AEs
Currie	Cheerleading	0.046	1000 AEs
Bonza	Mixed hs sports	0.023	1000 AEs
Kraeutler^*∗*^	Mixed sports	0.02	1000 AEs
Tee	Rugby	17	1000 hours
Junge	Rugby	9.45	1000 hours
Kawasaki	Boys rugby	8.5	1000 hours
Haesler	Rugby union	4.9	1000 hours
Gescheit	Boys tennis	3.6	1000 hours
Gescheit	Girls tennis	2.6	1000 hours
Junge	Soccer	0.21	1000 hours

^*∗*^describes shoulder dislocation injuries only.

**Table 6:** Incidence of shoulder injury stratified by the session type. Incidence rates by athletic exposure (AEs) have been standardized to 1 000 AEs and reported in descending order. If not stated otherwise, the sample population includes both girls and boys.

Author	Population	Competition	Practice	Units
Tuominen	Ice hockey	2	—	1000 AEs
Kerr	Boys football	1.65	0.25	1000 AEs
Lynall	Ice hockey	1.25	0.08	1000 AEs
Kroshaus	Wrestling	0.77	0.27	1000 AEs
Pierpoint	Boys lacrosse	0.55	0.09	1000 AEs
Bonza	Mixed hs sports	0.441	0.146	1000 AEs
Allen	Girls basketball	0.42	0.17	1000 AEs
Allen	Boys basketball	0.35	0.25	1000 AEs
Saper	Baseball	0.173	0.12	1000 AEs
Kerr	Boys soccer	0.15	0.03	1000 AEs
Clifton	Girls basketball	0.12	0.04	1000 AEs
Wasserman	Girls softball	0.12	0.09	1000 AEs
Clifton	Boys basketball	0.09	0.03	1000 AEs
DiStefano	Girls soccer	0.09	0.02	1000 AEs
Kerr	Girls volleyball	0.06	0.09	1000 AEs
Currie	Cheerleading	0.052	0.047	1000 AEs
Pierpoint	Girls lacrosse	0.05	0.01	1000 AEs
McCarthy^*∗*^	Mixed hs sports	0.0458	0.0086	1000 AEs

McCarthy et al. describes clavicle fracture injury types only, based on their injury definition.

**Table 7:** Assessment of study quality and level of evidence for included studies.

First Author	Risk of Bias (Downs and Black Score /33)	Level of Evidence
Achenbach	14	4
Allen	9	6
Archbold	8	4
Arnold	11	4
Asker	16	4
Asker	11	4
Badgley	7	6
Barden	9	4
Bonza	8	4
Bonza	10	3
Christine	9	6
Clarsen	7	4
Clifton	7	6
Clifton	7	6
Collins	7	6
Collins	7	6
Culpepper	11	4
Currie	9	6
Darrow	8	6
Deits	12	4
Deits	8	6
DiStefano	7	6
Dompier	9	5
Dyer	17	4
Emery	7	3
Finke	8	6
Fleisig	9	6
Fredriksen	20	2
Fredriksen (2021)	22	2
Frey	9	6
Gamage	9	4
Gescheit	9	4
Hibberd	13	3
Hinton	10	4
Hirschhorn	11	4
Hjelm	13	3
Hodhody	9	6
Horobeanu	10	4
Hutchinson	9	4
Jacobs	10	5
Junge	12	4
Kawasaki	12	6
Kerr (2017)	11	6
Kerr (2018, 2)	11	6
Kerr (2018, 3)	11	6
Kerr (2018)	11	6
Kraeutler	10	6
Krajnik	7	6
Kroshus	11	6
Lynall	11	6
Matic	7	6
McCarthy	8	6
Miranda	8	6
Moller	15	4
Morris	7	6
Myers	10	4
Oliver	7	6
Olsen	13	4
Oyama	12	4
Pasque	9	4
Pierpoint	11	6
Pierpoint	11	6
Polites	9	6
Pollard	12	4
Rauh	11	5
Reeser	8	4
Register-Mihalik	10	4
Robinson	9	6
Ruedl	10	4
Sakata	24	2
Saper	13	6
Schneur	8	6
Sewry	11	4
Shanley	7	4
Shanley	10	4
Shanley	9	4
Shanley	14	4
Shitara	9	4
Smith	8	4
Snyder-Vailer	7	6
Swenson	7	6
Tee (2017)	12	4
Tee (2017)	12	4
Tuominen	10	5
Tyler	10	4
Vijam	11	4
Volpi	12	4
Warner	9	6
Wasserman (2018)	11	4
Wasserman (2019)	11	5
Wood	7	6
Yard	13	4
Yard	13	4
Yard	9	4

## Data Availability

The datasets used and/or analyzed during the current study are available from the corresponding author on reasonable request.
